# Graphene Nanoparticle-Based, Nitrate Ion Sensor Characteristics

**DOI:** 10.3390/nano11010150

**Published:** 2021-01-09

**Authors:** Mohammad Taghi Ahmadi, Morteza Bodaghzadeh, Seyed Saeid Rahimian Koloor, Michal Petrů

**Affiliations:** 1 Nanotechnology Research Center, Nano-Physic Group, Physics Department, Urmia University, Urmia 5756151818, Iran; m.bodaghzadeh@gmail.com; 2 Institute for Nanomaterials, Advanced Technologies and Innovation, Technical University of Liberec, Studentská 2, 461 17 Liberec, Czech Republic; michal.petru@tul.cz; 3 School of Mechanical Engineering, Universiti Teknologi Malaysia, Johor Bahru 81310, Malaysia

**Keywords:** carbon nanoparticles sensor, carbon nanotube sensor, electrical discharge, water pollution, nitrate ions

## Abstract

Gathering and sensing of nitrate ions in the environment due to the abundant use in industry and agriculture have become an important problem, which needs to be overcome. On the other hand, new materials such as carbon-based materials with unique properties have become an ideal choice in sensing technology. In this research, the high-density polyethylene (HDPE) polymer as a carbon source in the melted form was used and carbon nanoparticles in the form of a strand between two electrodes were analyzed. It was fabricated between copper electrodes by the pulsed arc discharge method. Subsequently, the constructed metal–nanoparticle–metal (MNM) contact was employed to recognize the nitrate ions. Therefore, NaNO_3_, Pb(NO_3_)_2_, Zn(NO_3_)_2_, and NH_4_NO_3_ samples as a usual pollutant of industrial and agricultural wastewater were examined. All nitrate compounds in ten different densities were tested and sensor I-V characteristic was investigated, which showed that all the aforesaid compounds were recognizable by the graphene nano-strand. Additionally, the proposed structure in the presence of ions was simulated and acceptable agreement between them was reported. Additionally, the proposed structure analytically was investigated, and a comparison study between the proposed model and measured results was carried out and realistic agreement reported.

## 1. Introduction

Nitrate ions are widespread in the environment due to their high solubility in most liquids. These ions are known as water pollutants, which can be found in industrial and agricultural wastewater and have been considered to be an important threat to environmental waters [1,2]. The main source of nitrate ions produced by the human is non-organic fertilizers, food preservatives, reactive in explosive materials, glass industry, and other chemical processes. In surface water, the normal amount of nitrate ions is in the order of a few tens of ppm, but a high density of these ions has been produced by agriculture and urban wastewater [3]. Nitrate ion accumulation in drinking water can lead to serious and dangerous disease [4,5,6,7]. Up until now, different methods such as fluorescent spectroscopy, Raman spectroscopy, and chromatography have been utilized to find nitrate ions, but they need complicated instruments and are expensive [8,9,10,11,12,13,14,15,16]. However, current-voltage analysis as a sensing element in three or two-terminal devices with high sensitivity, requiring cheap facilities and proper for direct analysis, have been focused on [17]. After the discovery of nanoscale carbon-based materials [18], many studies have been carried out to use them in electronic devices [19,20]. According to their geometry, they can be conductive or semiconductor, which has diversified their application in diodes, transistors, and sensors [21,22,23,24]. A carbon-based sensor, which converts one physical phenomenon to an electrical signal, can be employed in identification systems [25,26,27,28,29]. Among the studies that have concentrated on the utilization of various nanoparticles in nitrate/nitrite detection sensors in the recent five years, K.R. Venugopala Reddy et al. (2020) employed cobalt (II) tetra methyl-quinoline oxy bridged phthalocyanine carbon nanoparticles [30,31,32,33,34,35,36,37,38,39], and Kattar Hanane et al. (2020) suggested tetradodecyl ammonium nitrate (TDAN) application [31]. Additionally, more sensors by Saad S. M. Hassan et al. (2019) Multi-walled carbon nanotubes (MWCNTs) have been investigated [32]; Lei Wu et al. (2018) employed Cu_2_O/CNT composites [33]; Yi Zhang et al. (2018) proposed a Ag/Cu/MWNTs/GCE platform [34]; Abdel Hameed et al. (2018) supported Cu@Pt/Gr nanoparticles on graphene configuration [35]; the Ghanei-Motlagh Taher (2018) Ag/HNT/MoS_2_ arrangement was configured [36]; Yue Wan et al. (2017) approved the application of a AgNP/MWCNT/GCE structure [37]; Bagheri et al. (2017) hired a Cu/MWCNT/RGO/GCE assembly [38]; and finally, Menart et al. (2015) worked on AgPs [39]. However, in this research, a graphene nanoparticle-based strand was fabricated by the pulsed arc discharge method between two metal electrodes. Due to the Fermi-level difference between the metal electrodes and graphene nanoparticle-based strand, in the shaped sensor, a Schottky-barrier was formed and examined. By adding a small amount of nitrate ion impurity to the sensor, the number of carriers varied and the I-V characteristic was altered, which led to electrical resistance variability in the strand, therefore, the resistance deviation in the nanoparticles was used in the measurement of physical phenomena. Its advantages over other nitrite reported sensors in the form of low manufacturing cost, fast production speed, and high efficiency can be highlighted.

## 2. Experimental Measurements

**Figure 1:**
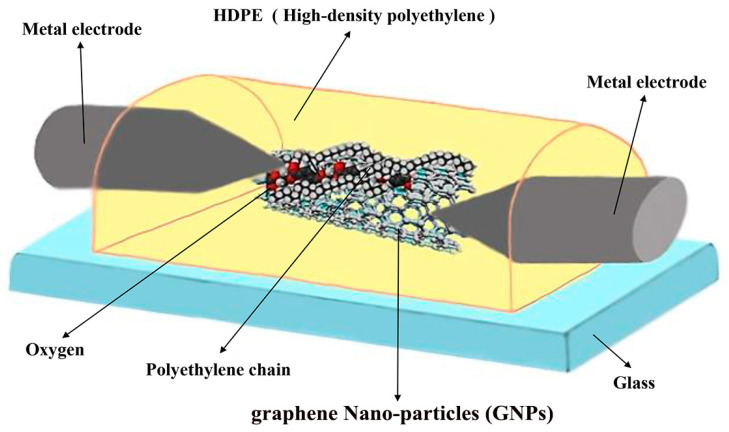
Schematic of fabricated graphene nanoparticle/high density poly ethylene (GNP/HDPE) composite between two metallic electrodes.

The entire experimental setup for carbon nanoparticle growth is presented in Appendix A; however, the schematic of the fabricated graphene nanoparticles (GNPs) in the melted high density poly ethylene (HDPE) composite is shown in Figure 1. A pulsed arc discharge method is employed in the presented work. In this mechanism, the voltage between two metal electrodes can be changed from 1–20 KV in low sequences.

**Figure 2:**
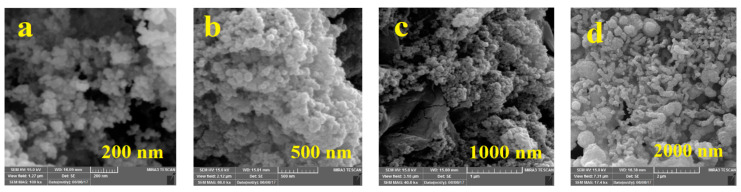

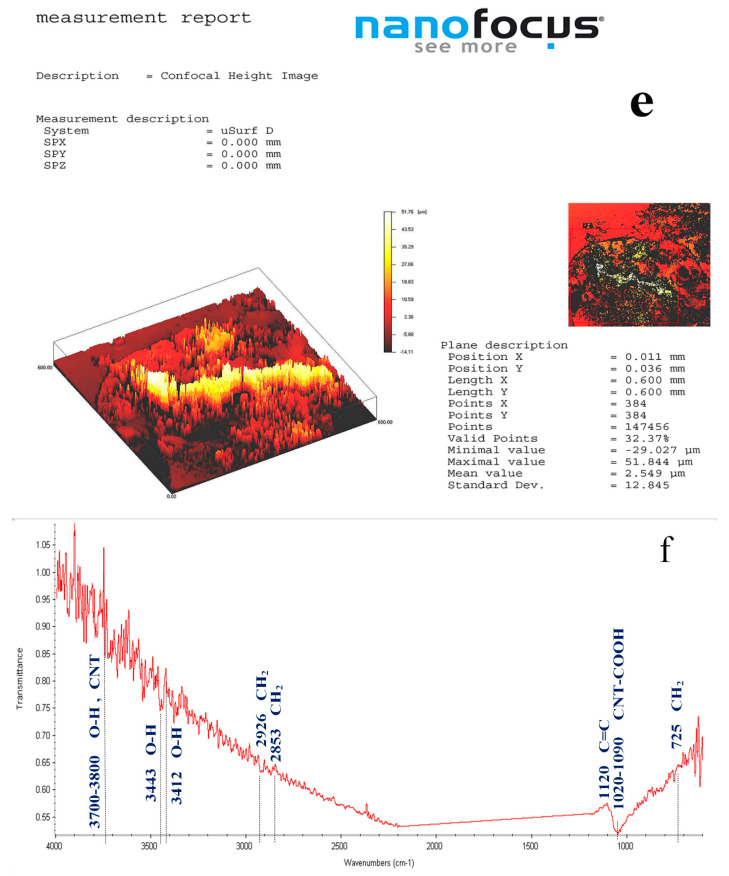
Scanning electron microscopy (SEM) photograph of Multi-walled carbon nanotubes/ high-density polyethylene (MWCNT/HDPE) nanocomposite sample. Imaging mode (**a**) ×100,000 at 15 KV, (**b**) ×60,000 at 15 KV, (**c**) ×40,000 at 15 KV, (**d**) ×17,400 at 15 KV and nano focus analysis of the sample (**e**) and Fourier transform infrared (FTIR) analysis of sample (**f**).

Two metal electrodes fixed on a glass substrate for easy nitrate ion injection into the sensor, hollow stainless-steel electrodes were employed. Three arrangements in the placement of electrodes on the substrate can be recognized such as plane to plane (PTP), tip to the plane (TTP), and tip to tip (TTT) configurations, which deepens the electrode shape. To increase the growth rate in the presented work, the TTT was employed. To synthesize stable carbon nanoparticles, melted high-density polyethylene (HDPE, (C_2_H_4_)_n_), due to a large number of carbon atoms in its chain, was used as a carbon source between the two electrodes. At atmospheric pressure, the HDPE was melted and placed between two electrodes. The high electric field graphene nanoparticles were synthesized between two electrodes within 24 s at the voltage of about 4.4 kV. Initially, the fabricated strand was analyzed by the nano-focus (Mahr Metering Systems GmbH, Göttingen, Germany) as shown in Figure 2e, which indicates the topological presence of carbon nanoparticles in the sensor. For a closer look, images of the scanning electron microscope (SEM) (TESCAN, Brno, Czech Republic) were taken as depicted in Figure 2a for 200 nm, Figure 2b for 500 nm, Figure 2c for 1000 nm, and Figure 2d for 2000 nm.

Among the various kinds of carbon nanoparticles, graphite, graphene, and CNTs, particularly the high electrical conductivity of CNTs, have been reported. Additionally, based on the results presented by [40,41,42] and a comparison of the scanning electron microscopy (SEM) images in Figure 2a–d with SEM images taken by [43], coated CNTs by HDPE can be concluded similarly to the composites presented in [43]. Furthermore, bundled multiwall carbon nanotubes (MWCNTs) coated by HDPE chains with a similar manufacturing process have also been explained in our previous works [43]. Moreover, the Fourier transform infrared (FTIR) analysis as shown in Figure 2f indicates the composite form of GNPs in HDPE. The peaks at 2926 cm^−1^ and 2853 cm^−1^ and 725 cm^−1^ signify the CH_2_ groups in hydrocarbons [43]. The peak at 3443 cm^−1^ [43] and 3412 cm^−1^ [44] in the Fourier transform infrared (FTIR) (JASCO, Easton, MD, USA) spectrum of the GNPs can be assigned to the O-H vibration in the carboxyl group and the peaks at 1020–1090 cm^−1^ can be assigned to CNT-COOH and peaks at 1120 cm^−1^ signify the C=C, CNT, and the backbone of carbon nanotubes and peaks at 3700–3800 cm^−1^ can be assigned to CNT, OH groups from the unbound [45]. Based on research in [43,44,45,46,47,48,49] and a comparison with the FTIR analysis taken from the sample, it can be concluded that the carbon nanoparticles grown also contained multiwall carbon nanotubes and GNPs.

After the growth of carbon nanoparticles between two electrodes, to examine their sensing phenomenon, solutions prepared in 10 different concentrations of four nitrate ions—NaNO_3_, Pb(NO_3_)_2_, Zn(NO_3_)_2_, and NH_4_NO_3_ in distilled water—for injection into the built sensor and the identification of nitrate ions tested. Therefore, the amount of nitrate ions in each solution was measured and prepared according to the minimum allowable amount of different nitrate ions in drinking water in the World Health Organization (WHO) guidelines [50]. First of all, NaNO_3_, as a common water pollutant from agricultural and industrial activities in 10 solutions was tested. To prepare the desired solution, the required amount of NaNO_3_ was measured and dissolved in distilled water. The 50 ppm, 100 ppm, 200 ppm, 500 ppm, 1000 ppm, 2000 ppm, 3000 ppm, 4000 ppm, and 5000 ppm solutions were selected.

**Figure 3:**
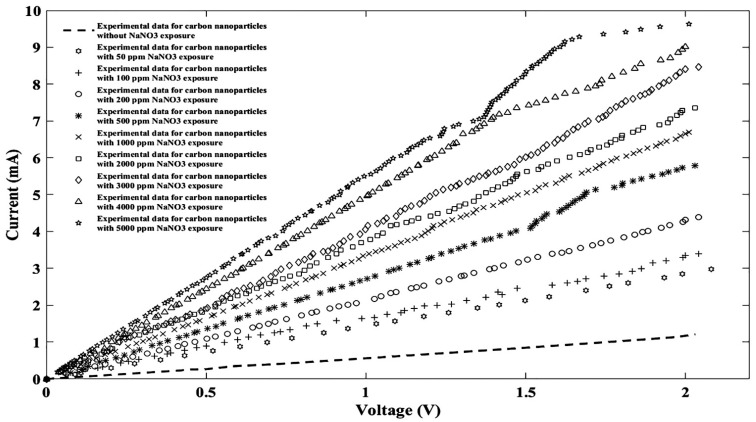
Current-voltage (I-V) characteristic of experimental data for the proposed sensor without/with nine different NaNO_3_ concentrations.

A current-voltage curve was taken from the fabricated sensor before ion injection and then each solution was injected into the device and subsequently an I-V curve was taken for each one. The I-V characteristic of the experimental data for this sensor without/with different NaNO_3_ concentrations is plotted in Figure 3, which indicates that the sensor current-voltage characteristic is affected by NaNO_3_ ion concentrations. The experimental results can be fitted by the Fourier model as:

f(x) = a_0_ + a_1_cos(xw) + b_1_sin(xw) + a_2_cos(2xw) + b_2_sin(2xw) + a_3_cos(3xw) + b_3_sin(3xw) + a_4_cos(4xw) + b_4_sin(4xw) + a_5_cos(5xw) + b_5_sin(5xw) + a_6_cos(6xw) + b_6_sin(6xw) + a_7_cos(7xw) + b_7_sin(7xw) + a_8_cos(8xw) + b_8_sin(8xw) (1)

where the fitting parameters (a_0–8_, b_1–8_, w) are calculated as shown in Table 1 for NaNO_3_.

**Figure 4:**
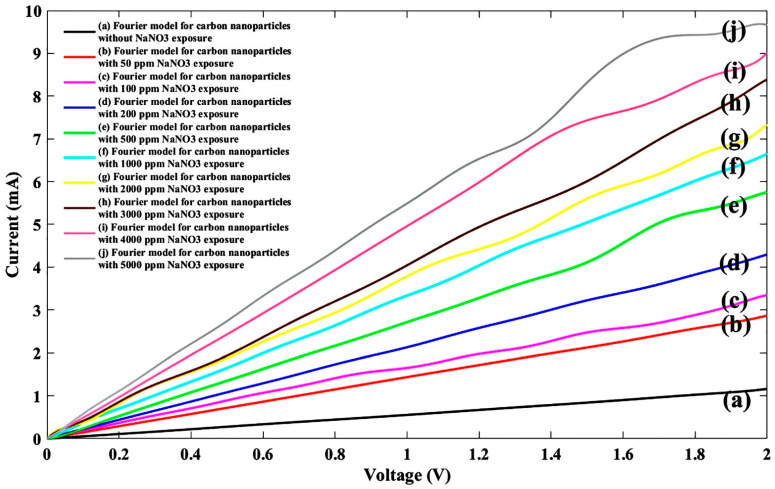
Fitted I-V characteristic of the experimental data with the Fourier model for the proposed sensor without NaNO_3_ exposure (**a**) and after injecting NaNO_3_ ions for nine different densities of (**b**) 50 ppm, (**c**) 100 ppm, (**d**) 200 ppm, (**e**) 500 ppm, (**f**) 1000 ppm, (**g**) 2000 ppm, (**h**) 3000 ppm, (**i**) 4000 ppm, and (**j**) 5000 ppm.

Based on the tabulated Fourier model (Table 1), the values for fitting parameters with corresponding regressions of NaNO_3_ exposure were re-plotted as shown in Figure 4.

**Figure 5:**
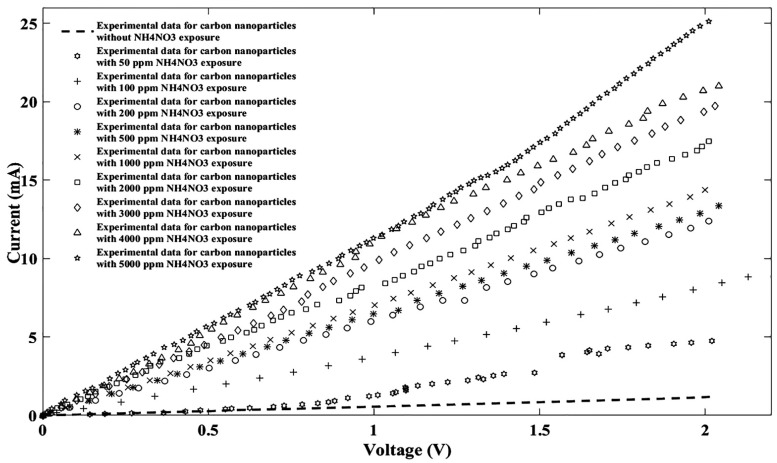
I-V characteristic of experimental data for the proposed sensor without/with ten different NH_4_NO_3_ concentrations.

Another common water pollutant, namely NH_4_NO_3_, has many applications in agricultural fertilizers and explosives production, is known as a nitrate ion source, and needs to be considered [38]. Therefore, ten NH_4_NO_3_ solutions prepared like the previous sample and injected to the sensitive region of sensor and sensor response to the NH_4_NO_3_ ions were plotted as shown in Figure 5.

To compare the efficacy of these ion concentrations on the sensor operation, fitting parameters for NH_4_NO_3_ were calculated from the Fourier model as shown in Table 2.

**Figure 6:**
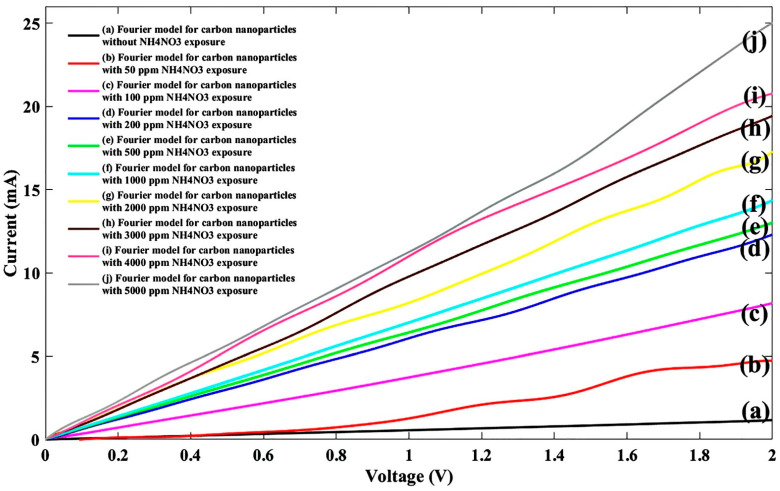
Fitted I-V diagram of experimental data with Fourier model for the proposed sensor without NH_4_NO_3_ exposure (**a**) and after injecting NH_4_NO_3_ ions for nine different densities (**b**) 50 ppm, (**c**) 100 ppm, (**d**) 200 ppm, (**e**) 500 ppm, (**f**) 1000 ppm, (**g**) 2000 ppm, (**h**) 3000 ppm, (**i**) 4000 ppm, (**j**) 5000 ppm.

When the fitting parameters for each concentration were calculated, the proper linear I-V characteristic was re-plotted as shown in Figure 6.

Like the sodium nitrate by concentration, the current also lifted on the ammonium nitrate (NH_4_NO_3_), and this similarity can be described by the ion concentration gradient.

**Figure 7:**
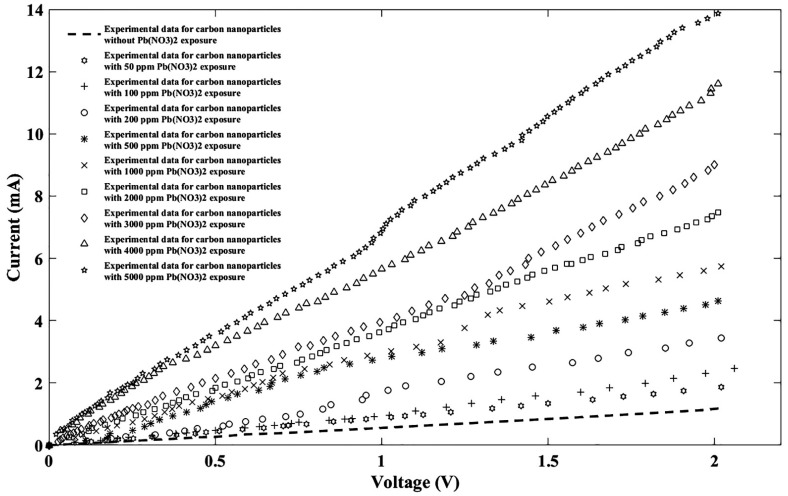
I-V diagram of experimental data for the proposed sensor without/with ten different Pb(NO_3_)_2_ concentrations.

Lead nitrate can be leaked to the environment from industrial activities, especially from battery manufacturing companies, which needs to be investigated carefully. Therefore, lead nitrate Pb(NO_3_)_2_ was tested with the same concentration as other nitrate ions, and the experimental response is plotted as shown in Figure 7.

Again, the Fourier model was used for fitting, and lead nitrate-related fitting parameters were carried out as shown in Table 3.

**Figure 8:**
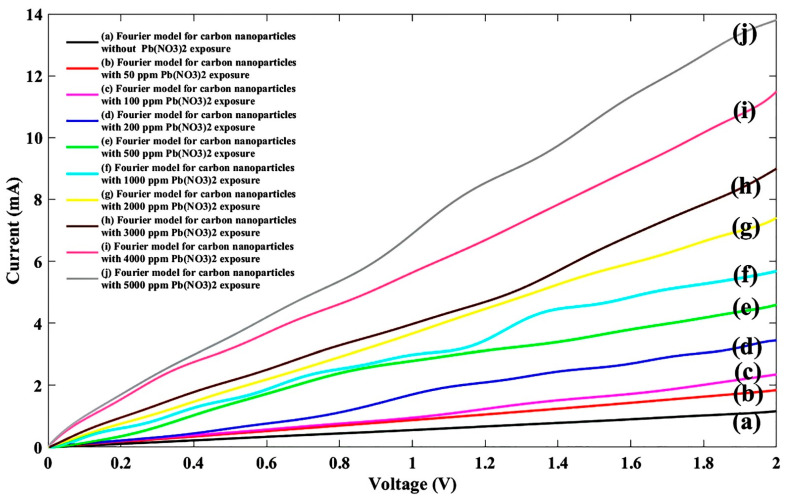
Fitted I-V diagram of the experimental data with the Fourier model for the proposed sensor without Pb(NO_3_)_2_ exposure (**a**) and after injecting Pb(NO_3_)_2_ ions for nine different densities of (**b**) 50 ppm, (**c**) 100 ppm, (**d**) 200 ppm, (**e**) 500 ppm, (**f**) 1000 ppm, (**g**) 2000 ppm, (**h**) 3000 ppm, (**i**) 4000 ppm, and (**j**) 5000 ppm.

To obtain the comparable scale in current-voltage relation according to the experimental outcomes, lead nitrate fitting parameters were employed and the I-V performance of the fabricated sensor was plotted as depicted in Figure 8.

**Figure 9:**
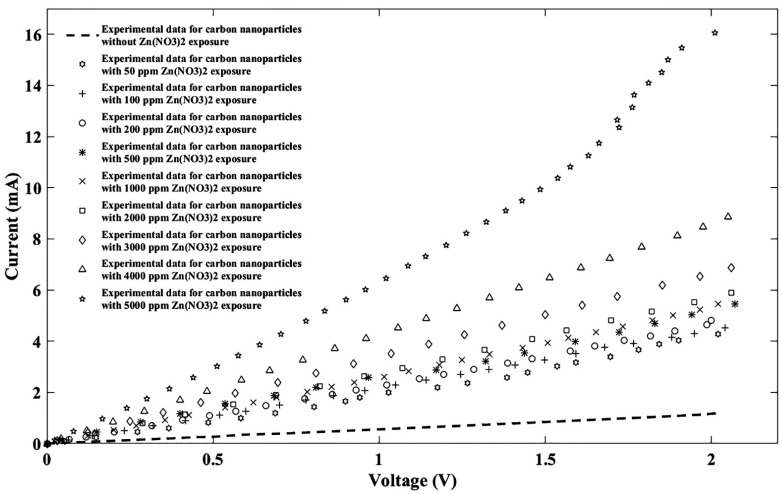
I-V diagram of experimental data for the proposed sensor without/with ten different Zn(NO_3_)_2_ concentrations.

Finally, ten solutions of zinc nitrate (Zn(NO_3_)_2_) were tested by the proposed sensor mechanism for 0 ppm, 50 ppm, 100 ppm, 200 ppm, 500 ppm, 1000 ppm, 2000 ppm, 3000 ppm, 4000 ppm, and 5000 ppm concentrations, the outcomes of which are reported in Figure 9.

In the zinc nitrate case, the best results on regressions were also obtained from the Fourier model, therefore the same fitting model was used and fitting parameters corresponding to the Zn(NO_3_)_2_ are tabulated in Table 4.

**Figure 10:**
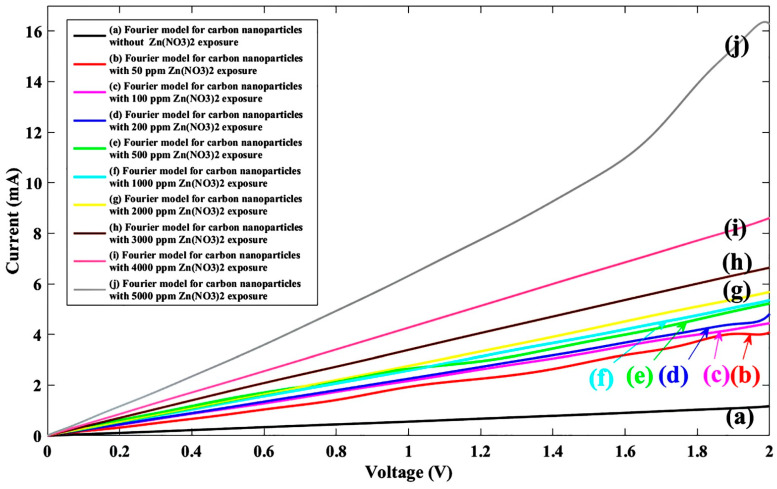
Fitted I-V diagram of experimental data with the Fourier model for the proposed sensor without Zn(NO_3_)_2_ exposure (**a**) and after injecting Zn(NO_3_)_2_ ions for nine different densities of (**b**) 50 ppm, (**c**) 100 ppm, (**d**) 200 ppm, (**e**) 500 ppm, (**f**) 1000 ppm, (**g**) 2000 ppm, (**h**) 3000 ppm, (**i**) 4000 ppm, (**j**) 5000 ppm.

As a final point, the Zn(NO_3_)_2_ corresponding fitted current-voltage characteristic was plotted and a sensor behavior assessment was carried out as shown in Figure 10.

Like the other nitrate ions under different ion concentrations, the variation in the current-voltage characteristic was also analyzed for the zinc nitrate solution. The solution concentration effect of the graphene nanoparticle-based sensor in the attendance of nitrate family was almost the same by other nitrate ions. In the other words, by increasing the solution concentration for all nitrate ions, the current was increased, as shown in Figure 4, Figure 6, Figure 8 and Figure 10, which can be described by the same ion creation in the sensing region. Additionally, a comparison study between different nitrate families indicated that current variation under sodium nitrate exposure was lower than other nitrates and ammonium nitrate demonstrated a larger variation in I-V characteristics. It can be deduced that the graphene nanoparticle strand illustrated more sensitivity to ammonium nitrate compounds.

## 3. Simulation Study

**Figure 11:**
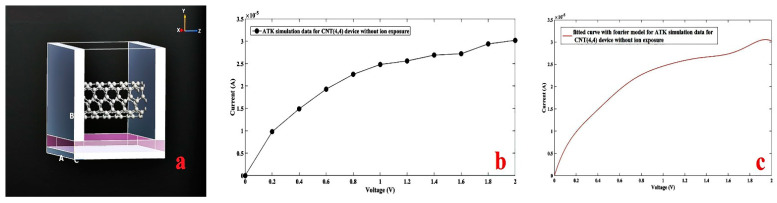
(**a**) Designed carbon nanotube field-effect transistor (CNTFET) for CNT (4,4), (**b**) simulated I-V characteristic without ion exposure, (**c**) Fourier fitted I-V characteristic without ion exposure.

Due to the limitations of the simulator, the simulation study could only be undertaken for the carbon nanotube-based sensors. Therefore, in the simulator, a carbon nanotube (CNT) with chirality (4,4) was positioned on top of a dielectric that was controlled by a metal gate under the dielectric. Ideally, nitrate ion impurities placed around the designed channel region and the device function in the attendance of nitrate ions were examined. First, a bare CNT semi-field effect transistor (CNTFET) was designed (Figure 11a) and its I-V characteristic was carried out in the original form (Figure 11b) and fitted form (Figure 11c) is reported in Figure 11.

In all samples, carbon nanotubes (4,4), which are a conductive nanotube, were used and the C–C bond length in all samples was selected as about 1.42086 A° and the repeated number for the nanotube length was set as C = 6. The lattice parameters were performed by the software itself for each given transistor and then fitted automatically to optimize the lattice. The left and right electrodes were considered to be metal, with a thickness of 2 A°. For each structure, two gates were placed under the simulated carbon nanotube. The metal gate was located at the bottom with a height of 1 A° and a voltage of 1 Volt was applied.

**Figure 12:**
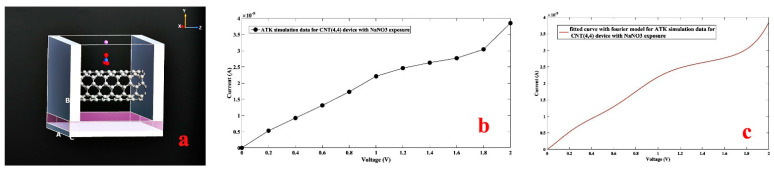
(**a**) Designed CNTFET for CNT (4,4) with attached sodium nitrate, (**b**) simulated I-V characteristic with NaNO_3_ exposure, (**c**) Fourier fitted I-V characteristic with NaNO_3_.

Initially, sodium nitrate was placed at the interaction with the carbon nanotube field-effect transistor (CNTFET) channel and the structure was optimized by the software Optimizer to allow all grafting to apply its effect on the channel region, and then the entire structure was relaxed. At room temperature, a voltage of 0–2 V was applied. Subsequently, the current-voltage feature was analyzed and fitted by MATLAB software as shown in Figure 12.

**Figure 13:**
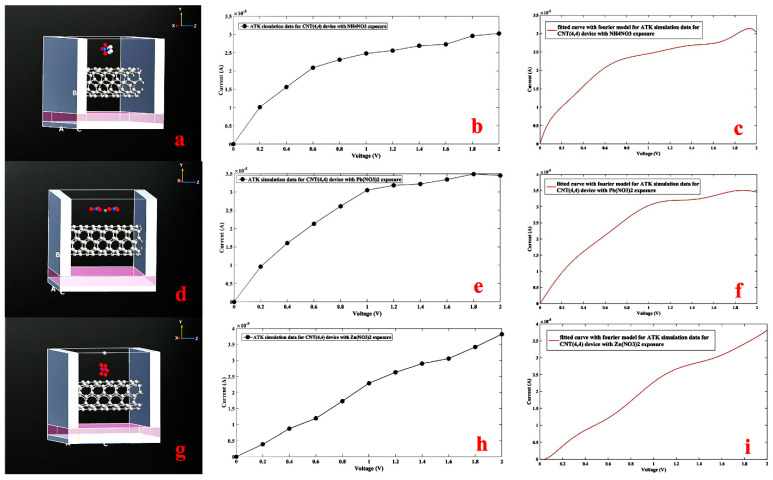
Designed CNTFET device for CNT (4,4), with NH_4_NO_3_ (**a**), Pb(NO_3_)_2_ (**b**) Zn(NO_3_)_2_ (**c**) exposure. simulated I-V curve with NH_4_NO_3_ (**d**), Pb(NO_3_)_2_, (**e**) Zn(NO_3_)_2_, (**f**) exposure. Fourier fitted I-V characteristic with NH_4_NO_3_ (**g**), Pb(NO_3_)_2_ (**h**), Zn(NO_3_)_2_(O) (**i**) exposure.

Similar to sodium nitrate, ammonium nitrate (Figure 13a–c), lead nitrate (Figure 13d–f), and zinc nitrate (Figure 13g–i) were proposed to the CNTFET channel at room temperature as shown in Figure 13.

## 4. Results and Discussion

**Figure 14:**
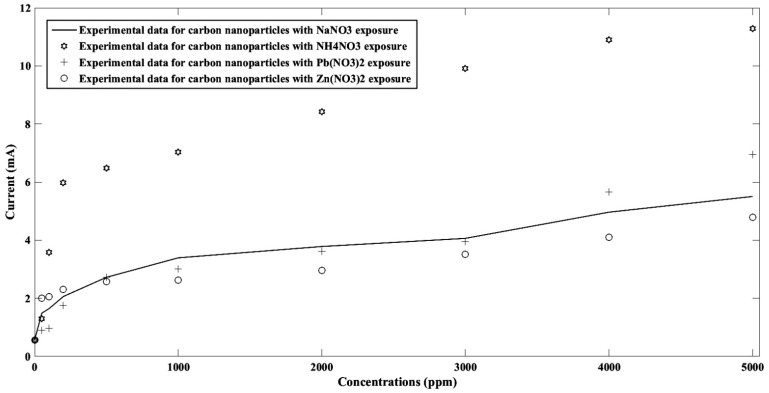
The relation of current-concentrations of the sensor at a specific voltage of 1 V for each of the NaNO_3_, Pb(NO_3_)_2_, Zn(NO_3_)_2_, and NH_4_NO_3_ ions in concentrations of 50 ppm, 100 ppm, 200 ppm, 500 ppm, 1000 ppm, 2000 ppm, 3000 ppm, 4000 ppm, and 5000 ppm.

To analyze the effect of analyte concentration on the fabricated sensor, in a certain voltage, the sensor responses for different pollutions were extracted from corresponding Figure 4, Figure 6, Figure 8 and Figure 10. Subsequently, its current at a specified voltage (1 V) for each pollutant, namely NaNO_3_, Pb(NO_3_)_2_, Zn(NO_3_)_2_, and NH_4_NO_3_ ions in concentrations of 50 ppm, 100 ppm, 200 ppm, 500 ppm, 1000 ppm, 2000 ppm, 3000 ppm, 4000 ppm, and 5000 ppm was calculated and plotted as shown in Figure 14. It can be concluded that by increasing the pollution concentration, the corresponding current was increased, which can be explained in the form of injected carrier increment in the sensitive region. It seems that all pollutants from this family follow the same trends on graphene-based metal–semiconductor–metal (MSM) structures.

**Figure 15:**
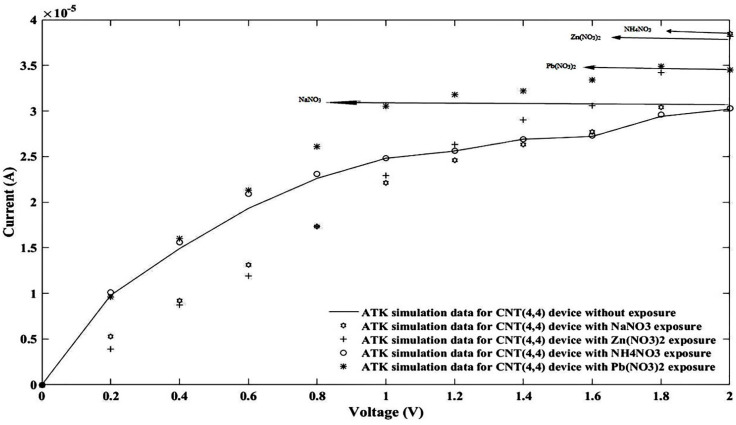
All I-V simulations for the CNTFET based sensor without/with nitrate ions.

Finally, to compare the simulation results, all simulated sensors on the FET platform were plotted in one graph as reported in Figure 15.

**Figure 16:**
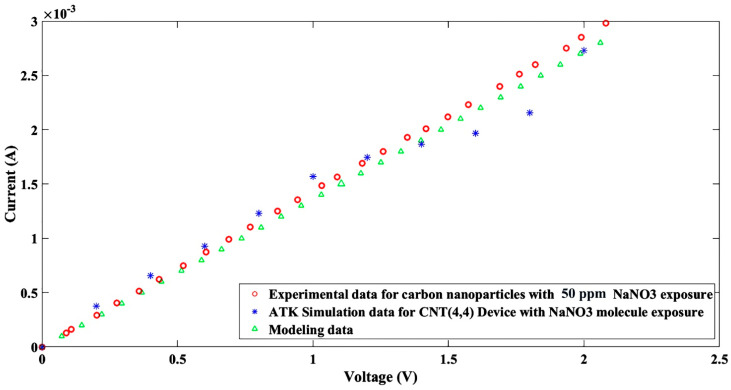
I-V characteristic curves for the experimental, simulation, and modeling data.

Since the simulated platform was not similar to the fabricated graphene nanoparticle-based sensor, therefore perfect agreement was not expected. However, for higher applied voltages, the higher current, the same as the fabricated device, was absorbed, which is related to the ammonium nitrate followed by zinc nitrate and lead nitrate; the lowest level current belonged to sodium nitrate. As the last point, the current–voltage model in the FET platform was tested with the sodium nitrate sensor as an example of a nitrate family sensor, and acceptable agreement between the FET regular model (Equation (2)) and the fabricated sensor is reported as shown in Figure 16.

(2) $$I\;=\;N_{c}F_{-\frac{1}{2}}\;(\eta ).\mathrm{qV}$$

where N_c_ is the effective density of state of graphene-based materials; q = 1.602 × 10^−19^ is an electron electric charge; $F_{-\frac{1}{2}}$ (η) is the Fermi integral of order (−1/2); and V is the carrier velocity [29]. Additionally, the proposed model was compared with the experimental and simulated results as shown in Figure 16, but a discrepancy in the different conditions was detected.

It can be concluded that the proposed model is accurate for an ideal case, but simulation results due to software boundary can be adopted for carbon-based devices. However, the fabricated device can be assumed as several parallel carbon nanoparticles-based devices, therefore, a discrepancy was observed with a minimum value of sodium nitrate, as shown in Figure 16.

## 5. Conclusions

Nitrate ions, as the main industrial and agricultural contaminations, are direct indicators on human health, therefore detection and gathering these ions from the environment are critical issues that need to be overcome. Moreover, new materials such as graphene nanoparticles in the application of this process have been encouraged. In this research, high-density graphene nanoparticles fabricated from a polyethylene polymer in strand form as a sensing environment was employed. Therefore, the current–voltage characteristic of metal–nanoparticle–metal (MNM) contact in the presence of nitrate ions such as NaNO_3_, Pb(NO_3_)_2_, Zn(NO_3_)_2_, and NH_4_NO_3_ was explored. Additionally, graphene nanoparticle-based sensor experimental results were compared with the simulation and theoretical model. It was concluded that sodium nitrate ions under identical applied voltage produce less current between nitrate families, which can vary from 0 up to 10 mA. The current variation under lead nitrate was about 0–14 mA and zinc nitrate indicated a current variation of about 0–16 mA, which is closer to the lead nitrate effect. However, the current deviation in the graphene strand for ammonium nitrate was 2.5 times greater than sodium nitrate, 1.79 times of lead nitrate, and 1.56 times than that of zinc nitrate. Therefore, a graphene nanoparticle strand structure as a sensing platform in the ionized medium is suggested.

## Figures and Tables

**Table 1:** Values for parameters of the Fourier model and the corresponding regressions for NaNO_3_ exposure.

General Model Fourier: f(x) = a_0_ + a_1_cos(xw) + b_1_sin(xw) + a_2_cos(2xw) + b_2_sin(2xw) + a_3_cos(3xw) + b_3_sin(3xw) + a_4_cos(4xw) + b_4_sin(4xw) + a_5_cos(5xw) + b_5_sin(5xw) + a_6_cos(6xw) + b_6_sin(6xw) + a_7_cos(7xw) + b_7_sin(7xw) + a_8_cos(8xw) + b_8_sin(8xw)										
	Without NaNO_3_	50 ppm NaNO_3_	100 ppm NaNO_3_	200 ppm NaNO_3_	500 ppm NaNO_3_	1000 ppm NaNO_3_	2000 ppm NaNO_3_	3000 ppm NaNO_3_	4000 ppm NaNO_3_	5000 ppm NaNO_3_
a_0_	2577	−4.365 × 10^9^	1.645	2.049	2.825	3.492	−6.718 × 10^10^	−7.474 × 10^4^	24.02	5.223
a_1_	−3251	6.984 × 10^9^	−0.7392	−1.475	−1.272	−1.534	1.079 × 10^11^	6.264 × 10^4^	−4.804	−2.429
b_1_	−2916	3.161 × 10^9^	−0.9671	−1.044	−1.855	−2.201	5.253 × 10^10^	1.172 × 10^5^	−36.7	−3.127
a_2_	283.3	−3.409 × 10^9^	−0.575	−0.7861	−1.003	−1.122	−5.268 × 10^10^	5.174 × 10^4^	−29.84	−1.644
b_2_	2608	−3.881 × 10^9^	−0.1857	0.267	−0.265	−0.4149	−6.713 × 10^10^	−7.71 × 10^4^	1.621	−0.4729
a_3_	624.7	7.667 × 10^8^	−0.3277	−0.1439	−0.4805	−0.6901	1.012 × 10^10^	−4.992 × 10^4^	0.7631	−0.8112
b_3_	−869.5	2.515 × 10^9^	0.1074	0.4591	0.2145	0.1724	4.668 × 10^10^	−5352	20.6	0.2365
a_4_	−261.9	1.259 × 10^8^	−0.1208	0.18	−0.1587	−0.2735	4.854 × 10^9^	7346	11.74	−0.4314
b_4_	57.75	−9.719 × 10^8^	0.1971	0.2272	0.2232	0.3322	−2.006 × 10^10^	1.899 × 10^4^	−0.3351	0.3087
a_5_	27.35	−1.329 × 10^8^	0.02184	0.1596	−0.01027	−0.0307	−4.223 × 10^9^	3940	−0.1503	−0.1108
b_5_	15.43	2.152× 10^8^	0.1266	−0.007545	0.1752	0.2552	5.149 × 10^9^	−4321	−5.373	0.3882
a_6_	0	3.356 × 10^7^	0.05205	0.04654	0.07374	0.07706	1.372 × 10^9^	−1017	−1.795	0.09396
b_6_	0	−2.263 × 10^7^	0.0422	−0.07136	0.06253	0.1278	−6.39 × 10^8^	−314.9	0.1281	0.1743
a_7_	0	−2.985 × 10^6^	0.03	−0.01211	0.02794	0.05692	−2.166 × 10^8^	8.98	0.06285	0.07132
b_7_	0	5.101 × 10^5^	0.01038	−0.03823	−0.01528	0.02353	−2.25 × 10^6^	92.4	0.3141	0.05917
a_8_	0	0	0.01524	−0.01777	0	0.03133	1.321 × 10^7^	0	0	0.05525
b_8_	0	0	−0.0199	0.0005103	0	−0.008749	6.461 × 10^6^	0	0	−0.001115
w	0.7738	0.4315	2.524	2.289	2.485	2.428	0.5159	1.027	1.76	2.592
R^2^	0.9998	1	0.9996	1	0.9998	0.9999	0.9998	0.9998	1	0.9998
SSE	0.0005779	0.0001708	0.0185	0.002341	0.04767	0.02591	0.08688	0.09976	0.03064	0.153
RMSE	0.006207	0.003941	0.02368	0.007057	0.02628	0.01823	0.03316	0.03329	0.01835	0.03764

**Table 2:** Values for fitting parameters from the Fourier model and its corresponding regressions for NH_4_NO_3_ exposure.

General Model Fourier: f(x) = a_0_ + a_1_cos(xw) + b_1_sin(xw) + a_2_cos(2xw) + b_2_sin(2xw) + a_3_cos(3xw) + b_3_sin(3xw) + a_4_cos(4xw) + b_4_sin(4xw) + a_5_cos(5xw) + b_5_sin(5xw) + a_6_cos(6xw) + b_6_sin(6xw) + a_7_cos(7xw) + b_7_sin(7xw) + a_8_cos(8xw) + b_8_sin(8xw)										
	Without NH_4_NO_3_	50 ppm NH_4_NO_3_	100 ppm NH_4_NO_3_	200 ppm NH_4_NO_3_	500 ppm NH_4_NO_3_	1000 ppm NH_4_NO_3_	2000 ppm NH_4_NO_3_	3000 ppm NH_4_NO_3_	4000 ppm NH_4_NO_3_	5000 ppm NH_4_NO_3_
a_0_	2577	1.907	−5.103 × 10^9^	6.266	6.715	6.618	−4.655 × 10^6^	7.381	11.73	11.23
a_1_	−3251	−0.5533	8.1 × 10^9^	−2.979	−4.666	−6.012	4.44× 10^6^	−10.42	−7.343	−8.179
b_1_	−2916	−2.046	3.832 × 10^9^	−3.901	−3.432	−2.714	7.164 × 10^6^	−1.082	−5.883	−6.337
a_2_	283.3	−0.7164	−3.829 × 10^9^	−2.174	−2.345	−2.283	2.773 × 10^6^	−2.319	−3.641	−4.802
b_2_	2608	−0.3164	−4.668 × 10^9^	−0.5321	0.7776	2.101	−5.586 × 10^6^	5.58	0.2512	1.815
a_3_	624.7	−0.4016	7.452 × 10^8^	−1.108	−0.3724	0.5924	−3.716 × 10^6^	2.811	−1.127	−0.6932
b_3_	−869.5	0.07183	2.98 × 10^9^	0.5151	1.196	1.732	3.543 × 10^5^	3.226	1.258	2.86
a_4_	−261.9	−0.1694	2.241 × 10^8^	−0.3338	0.4025	1.112	1.076 × 10^6^	2.83	−0.119	1.024
b_4_	57.75	0.06762	−1.123 × 10^9^	0.6515	0.4651	0.1004	1.409 × 10^6^	−0.4364	0.8636	1.368
a_5_	27.35	−0.1128	−1.762 × 10^8^	0.1117	0.2453	0.3058	2.282 × 10^5^	0.6937	0.3434	0.9994
b_5_	15.43	0.1618	2.372 × 10^8^	0.3757	−0.02259	−0.5612	−6.053 × 10^5^	−1.674	0.3403	0.1117
a_6_	0	0.04741	4.171 × 10^7^	0.1399	0.02285	−0.211	−1.678 × 10^5^	−0.4929	0.1789	0.3885
b_6_	0	0.07607	−2.222 × 10^7^	0.1197	−0.08791	−0.2558	3.396 × 10^4^	−0.813	0.03491	−0.3732
a_7_	0	0	−3.531 × 10^6^	0.09189	0	−0.1269	2.033 × 10^4^	−0.4073	0	0.05043
b_7_	0	0	1.672 × 10^5^	−0.01911	0	0.04976	2.13 × 10^4^	−0.01703	0	−0.2131
a_8_	0	0	0	0	0	0.005272	598.9	−0.07972	0	−0.02578
b_8_	0	0	0	0	0	0.03742	−2411	0.1107	0	−0.08339
w	0.7738	2.586	0.4214	2.403	2.129	2.125	1.032	2.102	2.044	2.291
R^2^	0.9998	0.9971	1	0.9998	0.9999	1	0.9998	1	0.9999	1
SSE	0.0005779	0.2887	0.0001825	0.09508	0.05321	0.000866	0.274	0.07641	0.295	0.1916
RMSE	0.006207	0.09978	0.004776	0.07962	0.05292	0.007137	0.07477	0.05224	0.0918	0.05089

**Table 3:** Values of the fitting parameters of the Fourier model and its corresponding regressions for Pb(NO_3_)_2_ exposure.

General Model Fourier: f(x) = a_0_ + a_1_cos(xw) + b_1_sin(xw) + a_2_cos(2xw) + b_2_sin(2xw) + a_3_cos(3xw) + b_3_sin(3xw) + a_4_cos(4xw) + b_4_sin(4xw) + a_5_cos(5xw) + b_5_sin(5xw) + a_6_cos(6xw) + b_6_sin(6xw) + a_7_cos(7xw) + b_7_sin(7xw) + a_8_cos(8xw) + b_8_sin(8xw)										
	Without Pb(NO_3_)_2_	50 ppm Pb(NO_3_)_2_	100 ppm Pb(NO_3_)_2_	200 ppm Pb(NO_3_)_2_	500 ppm Pb(NO_3_)_2_	1000 ppm Pb(NO_3_)_2_	2000 ppm Pb(NO_3_)_2_	3000 ppm Pb(NO_3_)_2_	4000 ppm Pb(NO_3_)_2_	5000 ppm Pb(NO_3_)_2_
a_0_	2577	−9.734 × 10^8^	−192.8	1.571	5.862	3.13	5.437	4.367	7.275	9.145
a_1_	−3251	1.461 × 10^9^	73.86	−0.9443	0.7388	−1.163	−1.114	−2.161	−2.69	−6.585
b_1_	−2916	9.46 × 10^8^	338.7	−1.085	−6.503	−1.938	−4.589	−2.807	−5.421	−6.735
a_2_	283.3	−5.082 × 10^8^	222.6	−0.5231	−4.109	−0.8636	−2.563	−1.751	−3.8	−6.326
b_2_	2608	−1.132 × 10^9^	−104.2	−0.06866	−3.632	−0.4929	−1.831	−0.1014	−1.165	1.942
a_3_	624.7	−1.054 × 10^8^	−83.05	−0.2765	−3.572	−0.5539	−2.153	−0.7292	−2.509	−0.4802
b_3_	−869.5	6.893 × 10^8^	−106.6	0.2509	1.209	−0.1498	0.6529	0.5688	1.685	5.391
a_4_	−261.9	2.008 × 10^8^	−34.67	−0.01229	−0.286	−0.354	−0.4248	−0.1055	−0.05805	3.412
b_4_	57.75	−2.268 × 10^8^	42.77	0.1986	2.28	0.1775	1.335	0.5735	2.125	1.687
a_5_	27.35	−9.469 × 10^7^	13.63	0.1012	0.9668	−0.1713	0.4745	0.1827	1.086	1.597
b_5_	15.43	2.661 × 10^7^	6.547	0.1031	0.6424	0.1267	0.5844	0.2369	0.7821	−1.619
a_6_	0	2.152 × 10^7^	0.4214	0.06874	0.4006	−0.0414	0.3186	0.1304	0.6822	−0.4063
b_6_	0	6.588 × 10^6^	−2.137	0.007466	−0.2295	0.1633	−0.03974	0.02699	−0.2572	−0.9014
a_7_	0	−2.098 × 10^6^	0	0.02841	−0.0006677	0.01672	0.02892	0.05148	0.08045	−0.3317
b_7_	0	−2.471 × 10^6^	0	−0.03931	−0.1262	0.0548	−0.07618	−0.02689	−0.3162	0.0615
a_8_	0	2.989 × 10^4^	0	−0.01232	0	0.003648	0	0	−0.04208	0
b_8_	0	2.194 × 10^5^	0	−0.01392	0	0.0655	0	0	−0.08274	0
w	0.7738	0.5184	1.245	2.495	2.003	2.516	2.132	2.356	2.175	1.911
R^2^	0.9998	1	0.9999	0.9996	0.9996	0.9999	0.9999	0.9999	0.9999	0.9999
SSE	0.0005779	1.203 × 10^5^	0.001487	0.01478	0.03313	0.01241	0.0406	0.04764	0.06905	0.158
RMSE	0.006207	0.001001	0.009354	0.03371	0.03715	0.02323	0.02717	0.03087	0.03285	0.04364

**Table 4:** Values for fitting parameters of the Fourier model and the corresponding regressions for Zn(NO_3_)_2_ exposure.

General Model Fourier: f(x) = a_0_ + a_1_cos(xw) + b_1_sin(xw) + a_2_cos(2xw) + b_2_sin(2xw) + a_3_cos(3xw) + b_3_sin(3xw) + a_4_cos(4xw) + b_4_sin(4xw) + a_5_cos(5xw) + b_5_sin(5xw) + a_6_cos(6xw) + b_6_sin(6xw) + a_7_cos(7xw) + b_7_sin(7xw) + a_8_cos(8xw) + b_8_sin(8xw)										
	Without Zn(NO_3_)_2_	50 ppm Zn(NO_3_)_2_	100 ppm Zn(NO_3_)_2_	200 ppm Zn(NO_3_)_2_	500 ppm Zn(NO_3_)_2_	1000 ppm Zn(NO_3_)_2_	2000 ppm Zn(NO_3_)_2_	3000 ppm Zn(NO_3_)_2_	4000 ppm Zn(NO_3_)_2_	5000 ppm Zn(NO_3_)_2_
a_0_	2577	129.5	2.106	5.493 × 10^10^	2.852	−1.08 × 10^8^	3.183	−1.369 × 10^8^	2.712 × 10^9^	−2.359 × 10^11^
a_1_	−3251	40.08	−1.696	−8.591 ×10^10^	−1.577	9.435 × 10^7^	−2.801	1.66 × 10^8^	−3.726 × 10^9^	3.699 × 10^11^
b_1_	−2916	−234.8	−0.9627	−4.728 × 10^10^	−1.581	1.999 × 10^8^	−1.246	2.148 × 10^8^	−3.254 × 10^9^	2.015 × 10^11^
a_2_	283.3	−181.1	−0.7006	3.718 × 10^10^	−1.103	1.012 × 10^8^	−0.851	2.335 × 10^7^	5.598 × 10^8^	−1.62 × 10^11^
b_2_	2608	−66.26	0.4566	5.871 × 10^10^	0.07772	−1.798 × 10^8^	0.917	−2.338 × 10^8^	3.68 × 10^9^	−2.509 × 10^11^
a_3_	624.7	−67.73	0.03618	−2.378 × 10^9^	−0.3309	−1.469 × 10^8^	0.2801	−1.025 × 10^8^	1.135 × 10^9^	1.243 × 10^10^
b_3_	−869.5	116.4	0.4209	−3.862 × 10^10^	0.3919	3.64 × 10^7^	0.4845	1.153 × 10^8^	−1.956 × 10^9^	1.66 × 10^11^
a_4_	−261.9	61.06	0.1922	−7.092 × 10^9^	0.02523	7.287 × 10^7^	0.2012	6.899 × 10^7^	−9.907 × 10^8^	2.94 × 10^10^
b_4_	57.75	51.31	0.09011	1.5 × 10^10^	0.2749	3.957 × 10^7^	−0.06968	−2.066 × 10^7^	4.143 × 10^8^	−6.517 × 10^10^
a_5_	27.35	29.6	0.07581	4.294 × 10^9^	0.08147	−1.199 × 10^7^	−0.01192	−2.204 × 10^7^	3.722 × 10^8^	−1.823 × 10^10^
b_5_	15.43	−25.25	−0.06483	−3.107 × 10^9^	0.06808	−3.061 × 10^7^	−0.05002	−4.96 × 10^6^	8.636 × 10^7^	1.382 × 10^10^
a_6_	0	−7.76	−0.01349	−1.18 × 10^9^	0.05804	−2.676 × 10^6^	0	3.251 × 10^6^	−6.438 × 10^7^	5.083 × 10^9^
b_6_	0	−12.73	−0.03419	1.466 × 10^8^	−0.001936	8.545 × 10^6^	0	2.794 × 10^6^	−7.057 × 10^7^	−7.723 × 10^8^
a_7_	0	−3.72	0	1.554 × 10^8^	0	1.239 × 10^6^	0	−1.41 × 10^5^	2.277 × 10^0^	−6.823 × 10^8^
b_7_	0	1.581	0	6.189 × 10^7^	0	−8.571 × 10^5^	0	−3.456 × 10^5^	1.477 × 10^0^	−2.467 × 10^8^
a_8_	0	0.1458	0	−7.082 × 10^6^	0	−1.185 × 10^5^	0	0	4.499 × 10^5^	3.216 × 10^7^
b_8_	0	0.5853	0	−8.59 × 10^6^	0	−5640	0	0	−1.006 × 10^6^	3.62 × 10^7^
w	0.7738	1.796	2.162	0.5236	2.13	0.6221	1.758	0.4357	0.5108	0.521
R^2^	0.9998	1	0.9999	1	1	1	1	1	1	0.9998
SSE	0.0005779	0.001893	0.001079	0.0003017	0.0007112	0.0002698	0.0002816	3.573 × 10^−5^	7.98 × 10^−5^	0.2171
RMSE	0.006207	0.01946	0.01039	0.006565	0.0154	0.005195	0.006851	0.002989	0.003647	0.1042

**Figure A1:**
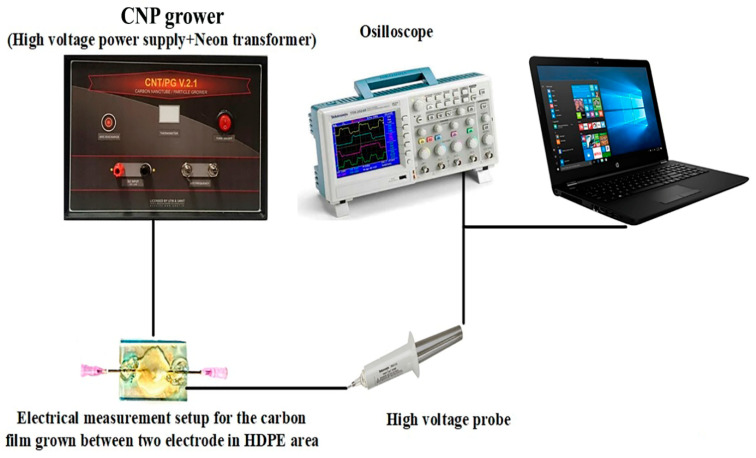
Complete experimental setup for GNP growth.

**Figure A2:**
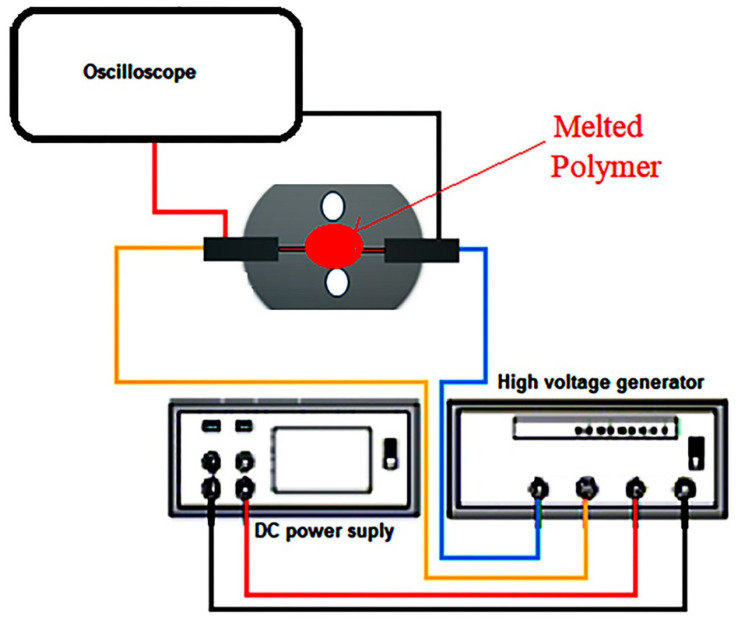
Experimental fabrication schematic diagram.

## Data Availability

Data available in a publicly accessible repository.

## Abbreviations

High-Density Polyethylene	(HDPE)
Nano-Particles	(GNPs)
Metal–Nanoparticle–Metal	(MNM)
Current-Voltage	(I-V)
Plane to Plane	(PTP)
Tip to Plane	(TTP)
Tip to Tip	(TTT)
Scanning Electron Microscope	(SEM)
Fourier-Transform Infrared spectroscopy	(FTIR)
Carbon Nanotubes	(CNTs)
Multiwall Carbon Nanotubes	(MWCNTs)
Ammonium Nitrate	(AN)
Carbon Nanotube Field Effect Transistor	(CNTFET)

## References

[1] Johnson C.J., Kross B.C. (1990). Continuing importance of nitrate contamination of groundwater and wells in rural areas. Am. J. Ind. Med..

[2] Wakida F.T., Lerner D.N. (2005). Non-agricultural sources of groundwater nitrate: A review and case study. Water Res..

[3] Alagha O., Manzar M.S., Zubair M., Anil I., Mu’Azu N.D., Qureshi A. (2020). Comparative Adsorptive Removal of Phosphate and Nitrate from Wastewater Using Biochar-MgAl LDH Nanocomposites: Coexisting Anions Effect and Mechanistic Studies. Nanomaterials.

[4] Brender J.D., Olive J.M., Felkner M., Suarez L., Marckwardt W., Hendricks K.A. (2004). Dietary Nitrites and Nitrates, Nitrosatable Drugs, and Neural Tube Defects. Epidemiology.

[5] Daniel W.L., Han M.S., Lee J.S., Mirkin C.A. (2009). Colorimetric Nitrite and Nitrate Detection with Gold Nanoparticle Probes and Kinetic End Points. J. Am. Chem. Soc..

[6] Greer F.R., Shannon M. (2005). Infant Methemoglobinemia: The Role of Dietary Nitrate in Food and Water. Pediatrics.

[7] Squillace P.J., Scott J.C., Moran M.J., Nolan B.T., Kolpin D.W. (2002). VOCs, Pesticides, Nitrate, and Their Mixtures in Groundwater Used for Drinking Water in the United States. Environ. Sci. Technol..

[8] Focazio M.J., Tipton D., Shapiro S.D., Geiger L.H. (2006). The Chemical Quality of Self-Supplied Domestic Well Water in the United States. Ground Water Monit. Remediat..

[9] Chon K., Lee Y., Traber J., Von Gunten U. (2013). Quantification and characterization of dissolved organic nitrogen in wastewater effluents by electrodialysis treatment followed by size-exclusion chromatography with nitrogen detection. Water Res..

[10] Kodamatani H., Yamazaki S., Saito K., Tomiyasu T., Komatsu Y. (2009). Selective determination method for measurement of nitrite and nitrate in water samples using high-performance liquid chromatography with post-column photochemical reaction and chemiluminescence detection. J. Chromatogr. A.

[11] Afkhami A., Madrakian T., Ghaedi H., Khanmohammadi H. (2012). Construction of a chemically modified electrode for the selective determination of nitrite and nitrate ions based on a new nanocomposite. Electrochim. Acta.

[12] Sachdeva V., Hooda V. (2014). A new immobilization and sensing platform for nitrate quantification. Talanta.

[13] Kim K., Kim K.L., Shin K.S. (2012). Selective detection of aqueous nitrite ions by surface-enhanced Raman scattering of 4-aminobenzenethiol on Au. Analyst.

[14] Ianoul A., Coleman A.T., Asher S.A. (2002). UV Resonance Raman Spectroscopic Detection of Nitrate and Nitrite in Wastewater Treatment Processes. Anal. Chem..

[15] Akyüz M., Ata Ş. (2009). Determination of low level nitrite and nitrate in biological, food and environmental samples by gas chromatography–mass spectrometry and liquid chromatography with fluorescence detection. Talanta.

[16] Marom H., Popowski Y., Antonov S., Gozin M. (2011). Toward the Development of the Direct and Selective Detection of Nitrates by a Bioinspired Mo–Cu System. Org. Lett..

[17] Anazawa K., Shimotani K., Manabe C., Watanabe H., Shimizu M. (2002). High-purity carbon nanotubes synthesis method by an arc discharging in magnetic field. Appl. Phys. Lett..

[18] Iijima S. (1991). Helical microtubules of graphitic carbon. Nat. Cell Biol..

[19] Ötvös Z., Onyestyák G., Hancz A., Kiricsi I., Rees L. (2006). Surface oxygen complexes as governors of neopentane sorption in multiwalled carbon nanotubes. Carbon.

[20] Chen L., Liu C., Liu K., Meng C., Hu C., Wang J., Fan S. (2011). High-Performance, Low-Voltage, and Easy-Operable Bending Actuator Based on Aligned Carbon Nanotube/Polymer Composites. ACS Nano.

[21] Balasubramanian K., Burghard M. (2005). Chemically Functionalized Carbon Nanotubes. Small.

[22] Marshall M.W., Popa-Nita S., Shapter J.G. (2006). Measurement of functionalised carbon nanotube carboxylic acid groups using a simple chemical process. Carbon.

[23] Karousis N., Tagmatarchis N., Tasis D. (2010). Current Progress on the Chemical Modification of Carbon Nanotubes. Chem. Rev..

[24] Bikiaris D., Vassiliou A., Chrissafis K., Paraskevopoulos K., Jannakoudakis A., Docoslis A. (2008). Effect of acid treated multi-walled carbon nanotubes on the mechanical, permeability, thermal properties and thermo-oxidative stability of isotactic polypropylene. Polym. Degrad. Stab..

[25] Zhou M., Lu Y.-H., Cai Y.-Q., Zhang C., Feng Y.-P. (2011). Adsorption of gas molecules on transition metal embedded graphene: A search for high-performance graphene-based catalysts and gas sensors. Nanotechnology.

[26] Wen X., Fan L., Yang S. (2004). Chemical assembly of nanostructured films for sensing applications. Nanosensing: Materials and Devices.

[27] Liu J.B., Chen J., Zhu L.F., She J.C., Deng S.Z., Xu N.S. Conductivity of screen-printed carbon nanotube composite film and its sensitivity to organic gas. Proceedings of the 2008 2nd IEEE International Nanoelectronics Conference.

[28] Akbari E., Buntat Z., Enzevaee A., Yazdi M.K., Bahadoran M., Nikoukar A. (2014). Sensing and identification of carbon monoxide using carbon films fabricated by methane arc discharge decomposition technique. Nanoscale Res. Lett..

[29] Neamen D.A. (2012). Semiconductor Physics, and Devices: Basic Principles.

[30] Jilani B.S., Malathesh P., Mruthyunjayachari C.D., Reddy K.V. (2020). Cobalt (II) tetra methyl-quinoline oxy bridged phthalocyanine carbon nano particles modified glassy carbon electrode for sensing nitrite: A voltammetric study. Mater. Chem. Phys..

[31] Hanane K., Messaoud B., Barhoumi H., Moncef T. (2020). Highly sensitive modified glassy carbon sensor based on TDAN for nitrate detection in real water. Mon. Chem.—Chem. Mon..

[32] SM Hassan S., Galal Eldin A., E Amr A.E.G., A Al-Omar M., H Kamel A., Khalifa N.M. (2019). Improved Solid-Contact Nitrate Ion Selective Electrodes Based on Multi-Walled Carbon Nanotubes (MWCNTs) as an Ion-to-Electron Transducer. Sensors.

[33] Wu L., Zhang X., Wang M., He L., Zhang Z. (2018). Preparation of Cu2O/CNTs composite and its application as sensing platform for detecting nitrite in water environment. Measurement.

[34] Zhang Y., Nie J., Wei H., Xu H., Wang Q., Cong Y., Tao J., Chu L., Zhou Y., Wu X. (2018). Electrochemical detection of nitrite ions using Ag/Cu/MWNT nanoclusters electrodeposited on a glassy carbon electrode. Sens. Actuators B Chem..

[35] Hameed R.A., Medany S.S. (2018). Sensitive nitrite detection at core-shell structured Cu@Pt nanoparticles supported on graphene. Appl. Surf. Sci..

[36] Ghanei-Motlagh M., Taher M.A. (2018). A novel electrochemical sensor based on silver/halloysite nanotube/molybdenum disulfide nanocomposite for efficient nitrite sensing. Biosens. Bioelectron..

[37] Wan Y., Zheng Y.F., Wan H.T., Yin H.Y., Song X.C. (2017). A novel electrochemical sensor based on Ag nanoparticles decorated multi-walled carbon nanotubes for applied determination of nitrite. Food Control..

[38] Bagheri H., Hajian A., Rezaei M., Shirzadmehr A. (2017). Composite of Cu metal nanoparticles-multiwall carbon nanotubes-reduced graphene oxide as a novel and high performance platform of the electrochemical sensor for simultaneous determination of nitrite and nitrate. J. Hazard. Mater..

[39] Menart E., Jovanovski V., Hočevar S.B. (2015). Silver particle-decorated carbon paste electrode based on ionic liquid for improved determination of nitrite. Electrochem. Commun..

[40] Moon Y.K., Lee J., Lee J.K., Kim T.K., Kim S.H. (2009). Synthesis of Length-Controlled Aerosol Carbon Nanotubes and Their Dispersion Stability in Aqueous Solution. Langmuir.

[41] Ding W., Eitan A., Fisher F.T., Chen X., Dikin D.A., Andrews R., Brinson L.C., Schadler L.S., Ruoff R.S. (2003). Direct Observation of Polymer Sheathing in Carbon Nanotube−Polycarbonate Composites. Nano Lett..

[42] Fadel T.R., Sharp F.A., Vudattu N., Ragheb R., Garyu J., Kim D., Hong E., Li N., Haller G.L., Pfefferle L.D. (2014). A carbon nanotube–polymer composite for T-cell therapy. Nat. Nanotechnol..

[43] Kasani H., Khodabakhsh R., Ahmadi M.T., Ochbelagh D.R., Ismail R. (2017). Electrical Properties of MWCNT/HDPE Composite-Based MSM Structure Under Neutron Irradiation. J. Electron. Mater..

[44] Ahmed D.S., Haider A.J., Mohammad M. (2013). Comparesion of Functionalization of Multi-Walled Carbon Nanotubes Treated by Oil Olive and Nitric Acid and their Characterization. Energy Procedia.

[45] Tucureanu V., Matei A., Avram A.M. (2016). FTIR Spectroscopy for Carbon Family Study. Crit. Rev. Anal. Chem..

[46] Celia E., De Givenchy E.T., Amigoni S., Guittard F. (2011). Three steps to organic–inorganic hybrid films showing superhydrophilic properties. Soft Matter.

[47] Cunha C., Panseri S., Iannazzo D., Piperno A., Pistone A., Fazio M., Russo A., Marcacci M., Galvagno S. (2012). Hybrid composites made of multiwalled carbon nanotubes functionalized with Fe3O4nanoparticles for tissue engineering applications. Nanotechnology.

[48] Stobinski L., Lesiak B., Kövér L., Tóth J., Biniak S., Trykowski G., Judek J. (2010). Multiwall carbon nanotubes purification and oxidation by nitric acid studied by the FTIR and electron spectroscopy methods. J. Alloys Compd..

[49] Goyanes S., Rubiolo G., Salazar A., Jimeno A., Corcuera M., Mondragon I. (2007). Carboxylation treatment of multiwalled carbon nanotubes monitored by infrared and ultraviolet spectroscopies and scanning probe microscopy. Diam. Relat. Mater..

[50] World Health Organization (2017). Guidelines for Drinking-Water Quality: Incorporating First Addendum.

